# Charge Regulation and pH Effects on Thermo-Osmotic Conversion

**DOI:** 10.3390/nano12162774

**Published:** 2022-08-13

**Authors:** Van-Phung Mai, Wei-Hao Huang, Ruey-Jen Yang

**Affiliations:** Department of Engineering Science, National Cheng Kung University, Tainan 70101, Taiwan

**Keywords:** thermo-osmotic conversion, ionic Seebeck coefficient, energy conversion, surface charge-regulation

## Abstract

Thermo-osmotic energy conversion using waste heat is one of the approaches to harvesting sustainable energy and reducing associated environmental impacts simultaneously. In principle, ions transport through a charged nanopore membrane under the effect of a thermal gradient, inducing a different voltage between two sides of the membrane. Recent publications mainly reported novel materials for enhancing the thermoelectric voltage in response to temperature difference, the so-called Seebeck coefficient. However, the effect of the surface charge distribution along nanopores on thermo-osmotic conversion has not been discussed yet. In this paper, a numerical simulation based on the Nernst–Planck–Poisson equations, Navier–Stokes equations, and heat transfer equations is carried out to consider the effect of surface charge-regulation density and pH of KCl solutions on the Seebeck coefficient. The results show that the highest ionic Seebeck coefficient of −0.64 mV/K is obtained at 10^−4^ M KCl solution and pH 9. The pH level and pore structure also reveal a strong effect on the thermo-osmotic performance. Moreover, the pH level at one reservoir is varied from 5 to 9, while the pH of 5 is fixed at the other reservoir to investigate the pH effect on the thermos-osmosis ion transport. The results confirm the feasibility that using the pH can enhance the thermo-osmotic conversion for harvesting osmotic power from low-grade heat energy.

## 1. Introduction

Harvesting energy for sustainable development has been one of the critical missions over the world. Solar and wind energy are being significantly developed and deployed on a large scale; however, their performance strongly depends on weather conditions [1]. Scientists are finding other resources that can convert energy from waste heat [2,3,4], for example, pressure-osmosis [5], droplet-triboelectric generators [6,7,8,9], and salinity gradient [10]. Thermo-osmosis conversion converts waste heat energy to electricity [11]. In principle, electrolytes containing positive and negative ions can be transported under a temperature gradient and surface-charge effect [12]. Using the temperature gradient, ions on the hot side with a higher diffusion are more active than that on the cold side [13]. Besides, a membrane containing a charged surface in its pore structure can control the direction and selectivity of ion transport [14,15]. The electrostatic force between positive ions and negative surface charge forms an electrical double layer (EDL). The EDL is a filter which attracts counter-ions and rejects a certain part of the co-ions to pass through the membrane [16]. Therefore, the surface charge in the membrane materials, pore size, and pore structure are the important parameters which decide the efficiency of thermo-osmosis. A 2D membrane with the layer-by-layer arrangement which forms high-density pore distribution of the nano-size channel has good potential application for thermo-osmosis conversion [17]. Sub-1 nm pore (with negative charge pore, for example) generates an overlapped EDL that only allows the positive ions to pass through the nano-channel and form the ion selectivity [18]. As a result, more positive ions are found on the cold side, and more negative ions remain on the hot side, leading to a different thermo-osmosis voltage.

The thermoelectric response in nanofluidics is involved with three sources, (1) thermo-electromigration [13,19], (2) Soret-type thermo diffusion [20,21,22,23], and (3) flow osmosis [24,25,26,27,28]. In thermo-electromigration, a thermoelectric potential is induced under the effect of the EDL in nanopores. Meanwhile, in the Soret-type thermo diffusion, the ions in the hot region of the nanopore diffuse naturally to the cooler side which generates thermoelectric potential as a result. Finally, flow osmosis is the process by which ions are driven by the flow field. The three sources exist simultaneously within the nanopore and are governed not only by the temperature gradient, flow field, and EDL but also by the interaction among them. The interaction of thermo-electromigration, thermo-diffusion, and flow osmosis is named the thermo-osmosis phenomenon.

Recent publications on the thermo-osmosis conversion reported the application of materials such as the covalent-organic framework [29], cellulosic membrane [30], and ionogels with cationic doping [31]. Others studied in thermo-osmosis showed hydrophilic or nanopore structures and membrane properties strongly influence the performance of the membrane [32,33]. Zhang et al. [34] discussed controlling ion transport through the nanopore by the surface charge of the 2D membrane. However, no study has been reported on the effect of pH environment or charge-regulation surfaces that may significantly control the ion transport in the confined sub-1 nm membrane used in thermo-osmosis energy conversion.

**Figure 1:**
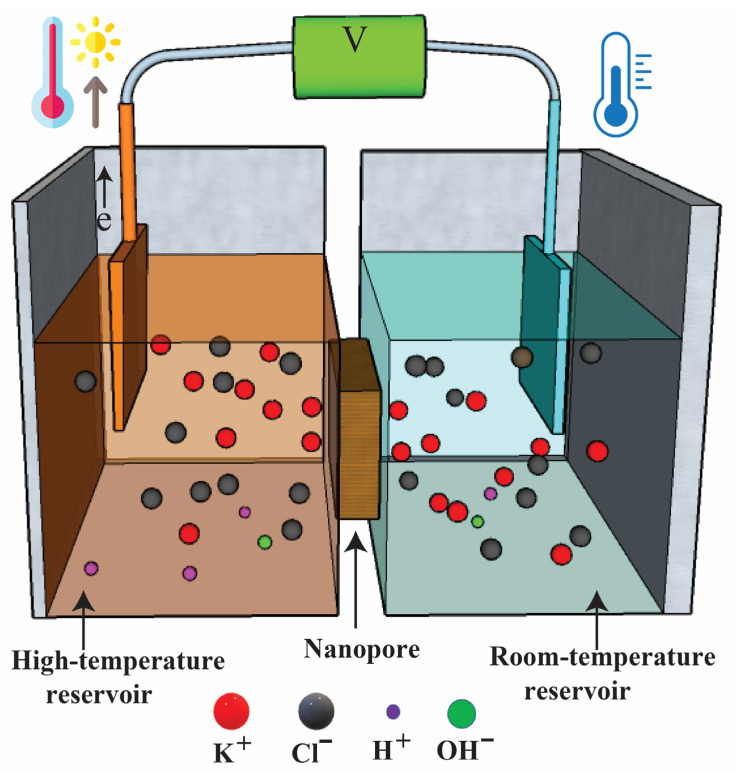
Illustration of thermal-osmotic generation using nanopore membrane.

In this paper, the feasibility of utilizing graphene oxide membrane to study the thermo-osmosis conversion is considered, and the ionic transport under a pH charge-regulated mechanism is discussed. Nanopore membranes fulfilled by a KCl solution are used to study the thermoelectric response (see Figure 1). Temperature-dependent ionic electromigration dominated the transport in the channel, and its effect becomes significant with decreases in the KCl solution concentration. Numerical simulation is carried out to explore the charge-governed effect on thermo-osmosis ion transport. A charge-regulation model and pH levels are adopted to describe the deprotonation and protonation processes [35]. Two cases of simulation settings are studied, including (1) the pH being constant in all computational domains to study the effect of the pH environment on the ionic Seebeck coefficient, (2) the pH 5 being fixed at one reservoir while the pH at another side is varied from 5 to 9 to explore the effect of the pH on the thermos-osmosis ion transport. The main purpose is to find an optimal operation condition that one can use to obtain the maximum ionic Seebeck coefficient.

## 2. Methodology

### 2.1. Theoretical Model and Mechanism

Ion transport under the effect of a temperature gradient and potential interaction were modelled using the coupling of the Nernst–Planck equation, Poisson equation, Navier–Stokes equations, and heat transfer equation. The governing equation for the *i*th ionic species flux was formulated as:

(1) $$J_{i}=c_{i}u-D_{i}\nabla c_{i}-\frac{F}{RT}D_{i}z_{i}c_{i}\nabla \phi -2\frac{D_{i}c_{i}\alpha_{i}}{T}\nabla T$$

(2) $$\nabla .J_{i}=0$$

(3) $$-\nabla .\left(\varepsilon_{r}\varepsilon_{0}\nabla \phi \right)=F\sum_{i=1}^{4}z_{i}c_{i}$$

where $\varepsilon_{0}$ is the permittivity of a vacuum, $\varepsilon_{r}$ is the relative permittivity, ϕ is the electric potential, and *F* is the Faraday constant. $z_{i}$ is the valence, $c_{i}$ is the concentration. $D_{i}$ is the diffusivity and $\alpha_{i}$ is the Soret coefficient. *i* denotes of the *i*th ionic specie including K^+^, Cl^−^, H^+^, and OH^−^. The inertial term in the Navier–Stokes equations was ignored in this study, which can be written as:

(4) $$-\nabla p+\nabla .\left(\mu \nabla u\right)-F\sum_{i=1}^{4}z_{i}c_{i}\nabla \phi -\frac{1}{2}\varepsilon_{0}{\left|-\nabla \phi \right|}^{2}\nabla \varepsilon_{r}=0$$

(5) $$\nabla .\left(\rho u\right)=0_{\;}$$

where *p*, ***u***, and *μ* are the pressure, flow field velocity and viscosity, respectively. The temperature in Equation (1) is obtained from the heat transfer equation as

(6) $$\rho C_{p}u.\nabla T=\nabla .\left(k\nabla T\right)$$

where ρ, *C_p_* and *k* are the density, specific capacity and thermal conductivity of the electrolyte, respectively. The ion diffusivity and viscosity of the electrolytes are temperature-dependent with the temperature in the range of 273 K to 373 K [10,14]. This range is smaller than that of solid materials for thermoelectric use [36]. Solid-state nanopore contains chemical functional groups which generate negative (or positive) surface charge density. The pH value strongly influences the deprotonation and protonation reactions of the functional group on the nanopore surface. The influence of the equilibrium deprotonation reaction that happened in functional carboxyl groups of graphene oxide was reported by Elisa et al. [37,38]. The surface charge density on the nanopore surface was modelled as [35,39,40]

(7) $$\sigma_{s}=\sigma_{0}\frac{10^{-pK_{A}}-10^{-pK_{B}}{\left[H^{+}\right]}_{s}^{2}}{10^{-pK_{A}}+{\left[H^{+}\right]}_{s}+10^{-pK_{B}}{\left[H^{+}\right]}_{s}^{2}}$$

where $\sigma_{0}$ is the basic charge density, *pK_A_* describes the acidity of −COOH functional group, *pK_B_* defines protonation reaction, and ${\left[H^{+}\right]}_{s}$ is the surface proton concentration.

### 2.2. Numerical Modelling

**Figure 2:**
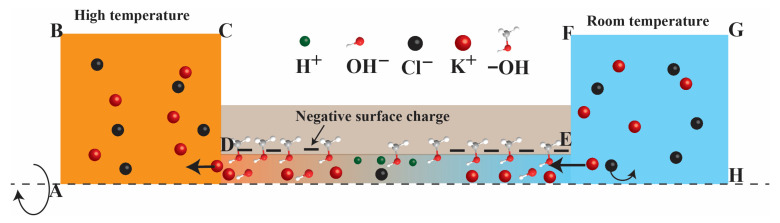
Illustration of charge-regulated surface controls thermo-osmosis.

The governing equations given above couple the relations between the ion transport, electrostatic field, flow field, and heat transfer modules. They are solved numerically by COMSOL Multiphysics simulations (COMSOL, Inc., Stockholm, Sweden). The purpose of the simulation is to investigate the combined effect of the thermal conditions and pH level in the surface charge regulation in KCl electrolyte within a confined nanopore (see Figure 2). The nanopore was assumed to have a size of 0.8 nm and the pore length was set as 50 nm. Moreover, the width and length of the two reservoirs were set as 1000 × 1000 nm^2^. The surface charge density on the nanopore wall was computed using Equation (7) with the basic charge ($\sigma_{0}$) set equal to −0.1 C/m^2^. The pH level was set in the range of 5 to 9. Finally, the asymmetric thermal (298 K and 298 + ΔT K) was set as the reservoir room temperature and the reservoir high temperature, respectively (See Table 1). Full details of the simulation process are also mentioned in our previous studies [10,14].

## 3. Results and Discussion

### 3.1. Verifying Numerical Simulation

**Figure 3:**
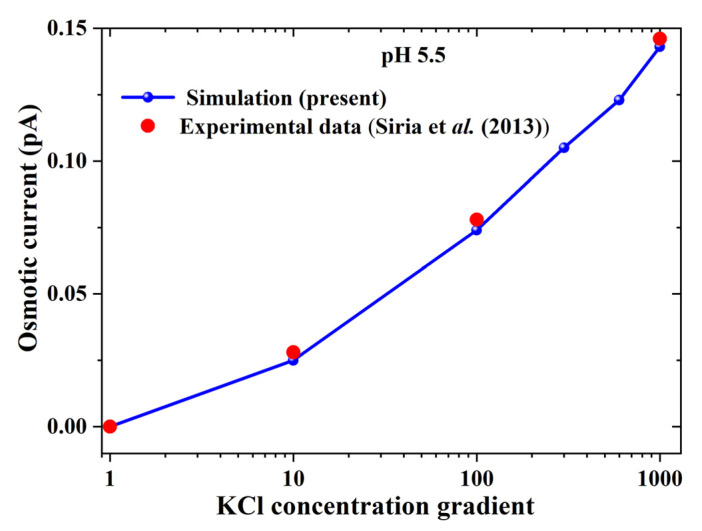
Comparison of present simulation and experimental results by Siria et al. [41] at pH 5.5.

To verify the numerical setting, the simulation results based on the coupled Poisson–Nernst–Plank and Navier–Stokes equations (PNP-NS) were first compared with the experimental data [41] (see Figure 3). The experiment used a boron nitride nanotube with a diameter of 40 nm and a length of 1250 nm. The results refer to a KCl solution with pH = 5.5, hence, four species (K^+^, H^+^, Cl^−^ and OH^−^) appeared in the system. The surface charge density is set as 18 sites/nm^2^ in accordance with the prediction of Siria et al. (2013) [41]. The pH condition used in the verification case will be applied in our simulation. Note that our simulation cases further explore the coupling effect of heat transfer and pH environment in ion transport.

### 3.2. Effect of Electrolyte Concentration on Ionic Seebeck Coefficient

**Figure 4:**
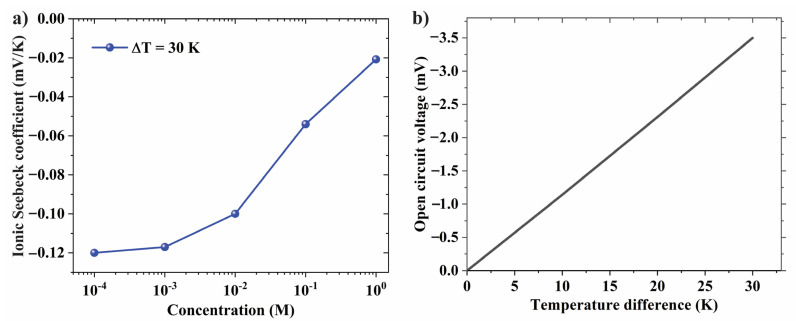
(**a**) Effect of electrolyte concentration on Seebeck coefficient. (**b**) Linear relation between open-circuit voltage and temperature difference.

Figure 4a shows the ionic Seebeck coefficient as a function of the concentration gradient. Note that the ionic Seebeck coefficient is defined as the ratio of voltage difference ΔV and temperature difference ΔT between the two ends of reservoirs as $S=-\frac{\Delta V}{\Delta T}$. As the concentration increases from 10^−4^ M to 10^−3^ M, the ionic Seebeck coefficient slowly goes down from −0.12 to −0.117. The ionic Seebeck coefficient noticeably drops as concentration further increases to 10^−2^ M. The reason is due to the overlapped electrical double layer (EDL) occurring in the confined space of the nanopore. As the concentration increases, the EDL thickness decreases, resulting in lower ion selectivity and more co-ions (e.g., Cl^−^) being transferred to the hot side compared to the overlapped EDL case. As a result, the thermos-electric voltage is reduced as the concentration increases. Therefore, we will choose the concentration of 10^−3^ M or 10^−4^ M for further analysis. Figure 4b shows the linear relationship between the open-circuit voltage and the temperature at the KCl concentration of 10^−4^ M and pH 5.5. The corresponding slope, which represents thermosensation selectivity, is obtained to be −0.12 mV K^−1^.

### 3.3. Effect of pH Level and Pore Structure on Ionic Seebeck Coefficient

**Figure 5:**
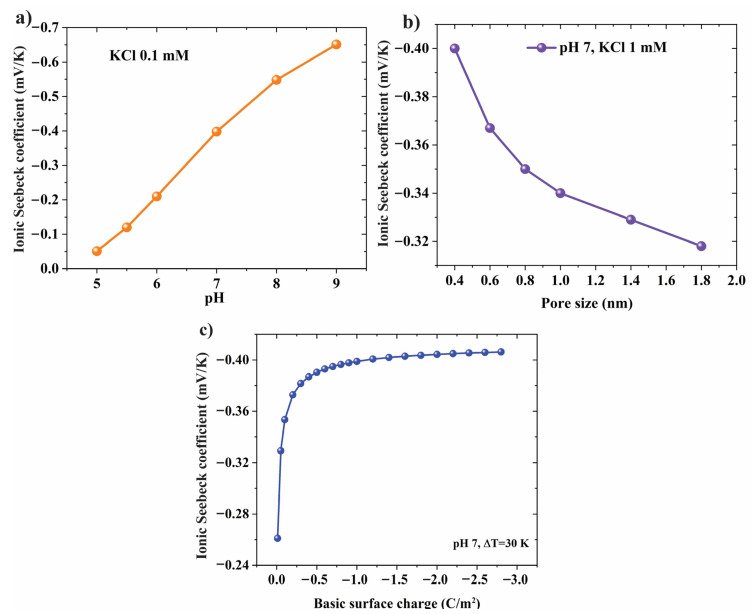
(**a**) Ionic Seebeck coefficient as functions of pH level, (**b**) pore size, and (**c**) basic surface charge.

Surface charge density has a significant impact on ion transport phenomena through nanopore membranes. The pH environment directly influences the deprotonation reaction on the surface of the membrane resulting in the change in the number of charge sites per nm^2^. Figure 5a shows the ionic Seebeck coefficient increases 13 times as the pH level increases from 5 to 9. Therefore, choosing high-surface charge materials and a high-pH environment are suggested to enhance thermoelectric performance. Pore or layer distance is also an important factor for boosting the Seebeck coefficient. Theoretically, ion transport in small pore size leads to the overlap of the EDL in nanochannels. Figure 5b shows that the ionic Seebeck coefficient decreases as pore size increases. Hence, using a confined membrane with sub-1 nm can take advantage of the confined space for thermo-osmotic ion transport. The thermos-electric performance is dramatically reduced as the layer distance or pore space increases. Figure 5c shows that the ionic Seebeck climbs remarkably as the magnitude of the surface charge increase from 0.01 to 0.3 C/m^2^, and then the ionic Seebeck saturates as the surface charge further increases.

### 3.4. Effect of pH Gradient on Thermo-Osmosis

**Figure 6:**
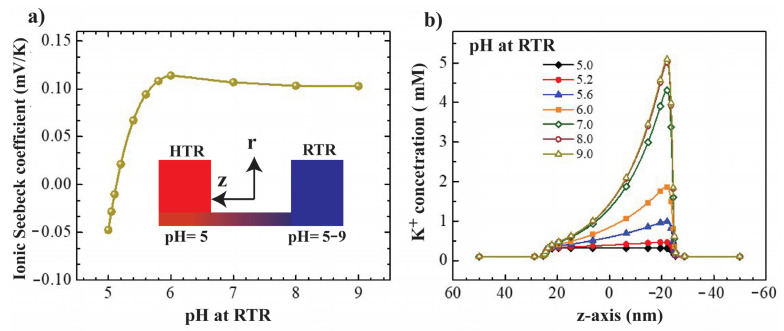
(**a**) Ionic Seebeck coefficient as a function of pH level at RTR. (**b**) K^+^ concentrations along the centreline of nanopore with the pH level at RTR varies from 5.0 to 9.0. Note that the pH level of HTR is fixed at 5.0.

To explore the effect of pH on thermo-osmotic ion transport in confined nanopore, simulations with different pH condition between two reservoirs are proposed. In case 1, the pH level at the high temperature reservoir (HTR) is fixed at 5.0, while the pH at the room temperature reservoir (RTR) is varied from 5 to 9. Figure 6a shows that the ionic Seebeck coefficient is changed from −0.05 to 0.1 as the pH level increases from 5.1 to 6.0 and then saturates as the pH level further increases from 6 to 9. A High pH level in the RTR induces more cation (e.g., K^+^) concentration gradient (see Figure 6b); however, it fails to increase the ionic Seebeck coefficient. To explain this saturation, we switch the pH level setting by fixing the pH 5 at RTR and varying pH at HTR, namely case 2.

**Figure 7:**
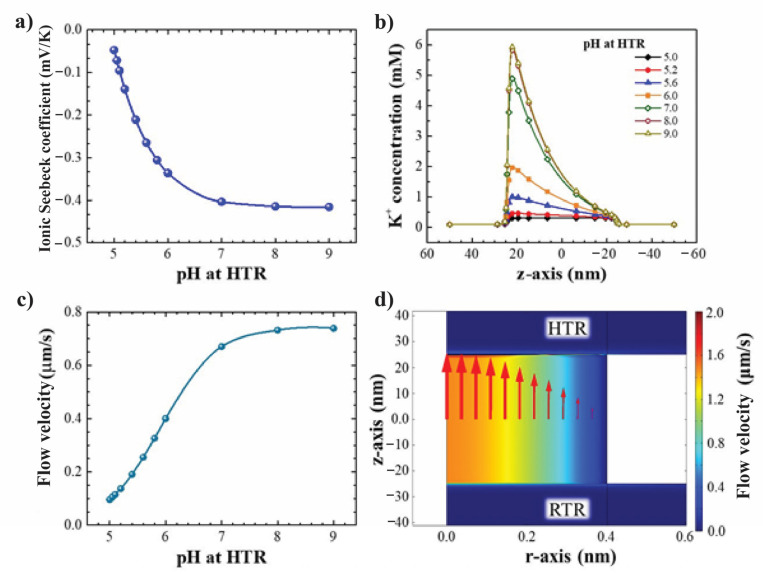
(**a**) Ionic Seebeck coefficient as a function of pH level at high-temperature reservoir. (**b**) K^+^ concentrations along the centreline of nanopore with respect to different pH levels at high-temperature reservoir. (**c**) Velocity of fluid flow as a function of pH level at HTR, the maximum velocity of 0.73 μm/s obtained at pH 9.0. (**d**) Flow field in nanopore (reservoirs are not in scale). Note that the pH level of RTR is fixed at 5.0.

In case 2, the pH level at the RTR was fixed at 5.0 while the pH level at the HTR varied from 5 to 9. Figure 7a shows that the magnitude of ionic Seebeck coefficient increases around eight times (from 0.05 to 0.415) as the pH level at the HTR increased from 5 to 7, and then slightly increases as the pH further increases to 9. Figure 7b shows the potassium concentrations along the centreline of nanopore. More K^+^ ion is attracted to the nanopore and then flows to the HTR as the high pH is set at the HTR. Moreover, the thermo-osmotic ion-transport is significantly enhanced, resulting in an increase in fluid flow through the nanopore from the RTR to the HTR (see Figure 7c,d). Overall, we explore that the effect of the pH on improving the ionic Seebeck coefficient in confined nanopore mainly contributed as a high pH level is set at the HTR.

## 4. Conclusions

In summary, we have demonstrated the thermo-osmostic conversion in a confined space. The effect of the pH environment on the charge-regulation material is examined. The results show that the highest ionic Seebeck coefficient of −0.64 mV/K is obtained at the concentration of 10^−4^ M KCl solution, pH 9, and it rapidly decreases as the concentration further increases. The improvement in the thermo-osmosis performance is mainly contributed by the charge-regulation under the effect of a high-pH environment or small confined space in nanopores. These findings confirm the feasibility of using pH in charge-regulation in the confined pore to enhance the thermo-osmosis performance.

## Figures and Tables

**Table 1:** Boundary conditions for axisymmetric model.

Surface	Electric Potential	Ion Transport	Flow Field	Heat Transfer
AB	Open circuit voltage	Concentration of K^+^, Cl^−^, H^+^, and OH^−^	Pressure = 0	298 + ΔT K
BC, FG	Zero charge n.∇ϕ=0	No flux $-n.J_{i}=0$	Slip	Thermal insulation
CD, EF	Zero charge n.∇ϕ=0	No flux $-n.J_{i}=0$	No slip	Thermal insulation
DE	Surface charge density $-\varepsilon_{0}\varepsilon_{r}\nabla \phi \cdot n=\sigma_{s}$	No flux $-n.J_{i}=0$	No slip	Thermal insulation
GH	Ground	Concentration of K^+^, Cl^−^, H^+^, and OH^−^	Pressure = 0	298 K

## Data Availability

Data will be provided via requests to the corresponding author.
